# Owners and Veterinary Surgeons in the United Kingdom Disagree about What Should Happen during a Small Animal Vaccination Consultation

**DOI:** 10.3390/vetsci5010007

**Published:** 2018-01-18

**Authors:** Zoe Belshaw, Natalie J. Robinson, Rachel S. Dean, Marnie L. Brennan

**Affiliations:** Centre for Evidence-based Veterinary Medicine, School of Veterinary Medicine and Science, University of Nottingham Sutton Bonington Campus, Leicestershire LE12 5RD, UK; z.belshaw.97@cantab.net (Z.B.); natalie.robinson@nottingham.ac.uk (N.J.R.); rachel.dean@nottingham.ac.uk (R.S.D.)

**Keywords:** preventative healthcare, dog, cat, veterinary, consultation, vaccination, qualitative, attitudes

## Abstract

Dog and cat vaccination consultations are a common part of small animal practice in the United Kingdom. Few data are available describing what happens during those consultations or what participants think about their content. The aim of this novel study was to investigate the attitudes of dog and cat owners and veterinary surgeons towards the content of small animal vaccination consultations. Telephone interviews with veterinary surgeons and pet owners captured rich qualitative data. Thematic analysis was performed to identify key themes. This study reports the theme describing attitudes towards the content of the consultation. Diverse preferences exist for what should be prioritised during vaccination consultations, and mismatched expectations may lead to negative experiences. Vaccination consultations for puppies and kittens were described to have a relatively standardised structure with an educational and preventative healthcare focus. In contrast, adult pet vaccination consultations were described to focus on current physical health problems with only limited discussion of preventative healthcare topics. This first qualitative exploration of UK vaccination consultation expectations suggests that the content and consistency of adult pet vaccination consultations may not meet the needs or expectations of all participants. Redefining preventative healthcare to include all preventable conditions may benefit owners, pets and veterinary surgeons, and may help to provide a clearer structure for adult pet vaccination consultations. This study represents a significant advance our understanding of this consultation type.

## 1. Introduction

Approximately one in three small animal consultations in the United Kingdom (UK) focuses on preventative healthcare, with a significant proportion of these consultations booked by the owner for the purpose of vaccination [1,2]. Since the 1950s, vaccination consultations have provided a source of consistent revenue for veterinary practices [3,4], but the content of such consultations has changed markedly. Initially, a clinical examination was performed at the time of vaccination to ensure the vaccine could safely be administered but the main focus remained on preventative healthcare. However, over time, veterinary surgeons identified that an annual health check examination had benefits for both animal health and practice profits [5] particularly when it identified health problems that owners had missed [6,7,8]. Preventative healthcare consultations in the UK are now more likely to include a full physical examination and discussion of a greater number of problems per pet than other consultation types [1,2]. 

There have been calls for the content of preventative healthcare consultations to be expanded further, particularly in relation to owner education about common preventable diseases and welfare problems. Hoskins [8] suggested owners should be provided with educational leaflets at the end of each adult dog vaccination consultation and Miller [9] advocated inclusion of “counselling on behavioural, nutritional and dental care” in addition to vaccination and parasite prevention. However, Shaw and colleagues found educational discussion to form only a small component of wellness consultations in Canada [10], with less than 1% of statements in observed consultations relating to anticipatory guidance. The time spent on educational topics in UK vaccination consultations has not been reported but the ongoing need for owner education in the UK is supported by results from the recent People’s Dispensary for Sick Animals Animal Wellbeing (PAW) survey [11]. This suggests many UK pet owners fail to provide adequate nutrition, exercise, company and preventative healthcare for their pets. Furthermore, behavioural problems, many of which are likely to be preventable, have been identified as the most common cause of euthanasia in dogs under 3 years of age in the UK [12]. The detrimental impact of these preventable conditions on the quality of life of pets [13] and their owners [14,15,16] are increasingly recognised as important aspects of One Welfare [17]. 

Preventative healthcare consultations in the United Kingdom are not significantly longer than consultations focused on a specific health problem [18], and these consultations are already under significant time pressure [19] without inclusion of additional material. Pet healthcare plans, increasingly common in the UK, have been suggested as a solution to this time pressure [4]. In these plans, the vaccination consultation is packaged in a monthly direct debit payment scheme with parasiticides, and frequently some consultations. Such plans are primarily aimed at increasing owner compliance with preventative medicine administration, with additional benefits of generating a regular monthly income for the practice. As the plans are predominantly administered by reception staff, Ravetz [4] suggests such plans also save time by limiting veterinary surgeons’ need to discuss preventative healthcare in the consulting room. However, to date attitudes towards content of preventative healthcare consultations have not been explored. Thus, it is not known whether these recent changes reflect the wishes or expectations of UK owners and veterinary surgeons. 

The aim of this study was to investigate owner and veterinary surgeon attitudes to the vaccination consultation using a qualitative methodology. The objective was to perform semi-structured interviews to discuss recent owner and veterinary surgeon experiences of vaccination consultations and to identify key emergent themes using thematic analysis.

## 2. Materials and Methods 

This study form part of a larger body of work, the aims of which were to ascertain the expectations, opinions and experiences of veterinary surgeons and owners were in relation to preventive medicine consultations in the UK, and to understand their perspectives on what could be done to maximise the benefits of preventive medicine consultations. Data were gathered through telephone interviews during July and August 2016 with dog and cat owners and veterinary surgeons conducting small animal consultations in the UK. The study was conducted in accordance with the Declaration of Helsinki and ethical approval was granted by the ethics committee at the School of Veterinary Medicine and Science, University of Nottingham (Reference number: 1521 150813). Reporting followed the Consolidated Criteria for Reporting Qualitative Research (COREQ) checklist [20].

### 2.1. Owner and Veterinary Surgeon Recruitment 

Recruitment of both owners and veterinary surgeons was based on separate purposive sampling frames designed by the authors and available on request. The aim of these sampling frames was to capture the maximum range of experiences of preventative healthcare consultations, as described by Bryman [21]. The owner sampling frame included pet, owner and practice variables, whilst the veterinary surgeon sampling frame included veterinary surgeon and practice variables.

Inclusion criteria for owner interviewees were: (a) ownership of one or more cat and/or dog that had attended a veterinary consultation in the UK during the preceding 3 months for any form of preventative healthcare consultation. Eligible consultation types were: routine vaccination; antibody titre testing; parasite prevention; routine health check; or (in female dogs) prevention of season; AND (b) willingness to be interviewed by telephone about that consultation during the study period. Inclusion criteria for veterinary surgeon interviewees were: (a) individuals currently working in a general practice in the United Kingdom; AND (b) who currently performed preventative healthcare consultations including dogs and/or cats; AND (c) who were available for telephone interview during the study period.

Owner recruitment was conducted using: direct contact to known eligible participants in the authors’ networks; posts on social media including online owner forums; recruitment of eligible clients by veterinary surgeons in a multi-branch veterinary practice in Scotland; and snowball sampling whereby recruited interviewees identify other potential interviewees [21]. Veterinary surgeon recruitment was conducted using: social media; by contacting veterinary practices who had expressed an interest in collaboration with the Centre for Evidence-based Veterinary Medicine in research; direct emails to the Royal College of Veterinary Surgeons’ list of practices; and snowball sampling. For the purpose of this study, probable data saturation was defined as the point at which no further significant variability emerged as a result conducting further interviews. This was then confirmed during thematic analysis (see below).

### 2.2. Interview Procedure

Owners and veterinary surgeons who expressed an initial interest in participation were emailed information about the study and a copy of the consent form. Those willing to proceed were asked to supply information relevant to the inclusion criteria and a date was then arranged for the interview. Incentives to participate were not provided. All interviews were conducted by telephone by author NR, a veterinary surgeon with a background in investigating preventative healthcare consultations who had received training in qualitative research methods. At the start of each interview, NR confirmed that the consent form had been read and understood, and asked whether there were any queries. Verbal consent to proceed was given by interviewees before the Dictaphone was started. Using data from previous studies and literature reviews to identify important topics, the authors developed separate semi-structured interview guides for owner and veterinary surgeon interviews (see Tables S1 and S2 in supplementary materials). These had been piloted with two veterinary surgeons and two owners, and minor changes to question order were made based on their feedback. Experiences with all preventative healthcare consultations during the preceding 3 months were discussed with the owners, and attitudes to preventative healthcare consultations in general were discussed with veterinary surgeons. In addition, owners and veterinary surgeons discussed their attitudes towards what happened, or should, happen during a preventative healthcare consultation.

### 2.3. Data Analysis

Interviews were recorded using a Dictaphone with a telephone adapter and were professionally transcribed verbatim. Transcripts were not returned to interviewees and repeat interviews were not performed. Data analysis was performed by ZB, a veterinary surgeon with previous experience of conducting qualitative research and analysing and reporting qualitative data [22]. Others were not involved in the analysis process due to challenges associated with the relevance of inter-rater reliability as described by Morse [23]. Transcripts were checked for accuracy against the recordings. Thematic analysis was performed following the six-step plan described by Braun and Clarke [24] using the organisational support of nVivo (nVivo qualitative data analysis software v11; QSR International Pty Ltd., Melbourne, Australia). Themes were identified using both inductive and deductive approaches, and analysis used a critical realist ontology. Data saturation was defined as the point at which no additional themes emerged as a result of analysing further transcripts. Statistical analysis was not performed as is standard for qualitative studies which aim to capture the diversity of experiences [25,26].

## 3. Results

Thirty-one interviews were arranged, but two owners were unavailable to participate on the day of the interview due to unforeseen circumstances and those interviews could not be rearranged during the time available. Twenty-nine telephone interviews were therefore completed, 14 with veterinary surgeons and 15 with owners. Demographic details collected for each interviewee are presented in supplementary materials (Tables S3 and S4). The veterinary surgeons, ten female and four male, were from 12 practices. Practice types included both corporate and independent, small animal only and mixed, single and multi-branch. All veterinary surgeons had graduated within the preceding 20 years and ranged in seniority from assistant to clinical director. Thirteen dog owners, one cat owner, and one dog and cat owner were interviewed. Dogs included pets, agility dogs and working gun dogs; cats lived both indoors and outdoors. Interview lengths ranged from 15 to 59 min (median 28 min; interquartile range 21–40.5 min). All participants answered all questions posed. Thematic analysis inductively identified four overarching themes. The theme relating to the expectations of both owners and vets as to what would happen during a vaccination consultation is reported below with illustrative quotes.

### 3.1. Owners

Owners identified expectations both about the content of their pet’s vaccination consultation and of the experience of attending that consultation. None referred to any information from their veterinary practice about what would be covered during a vaccination consultation or what they should do in preparation for attending that consultation. As a result, expectations were based primarily on direct experience of previous consultations; owners who had not previously attended a vaccination consultation did not know what they should have expected. Experienced owners identified that vaccination consultation content could be highly variable within the same practice. Typically, the greatest source of this variability was considered to be the veterinary surgeon, particularly in relation to their communication style, the pace of the consultation and the thoroughness of the clinical examination. For this reason, several owners suggested a checklist should be used by veterinary surgeons to improve the consistency of the experience.

“*After he gave him the injection, as a kind of afterthought, he sort of lifted him up on the table and checked his testicles. He didn’t tell me why he was doing that. Obviously I knew. But he didn’t tell me why he was doing that or…or anything. So it was very different. It was really quick this one, you know.*”

[Owner 10]

Consequently, several discussed the importance to them of the vaccination consultation being conducted by a specific veterinary surgeon with whom they were familiar. Rarely, owners described changing veterinary surgeons or even practices until they found one that matched their expectations.

“*I like to have that relationship that I know the vet, trust the vet. I feel that they then have an appreciation of what I’m doing with my dogs and my expectations of my dogs and things. And I like to think it’s a mutually, not beneficial, but sort of a mutually respectful relationship on that basis.*”

[Owner 5]

Many owners thought that discussion about preventative healthcare should be the main focus of this consultation. Conversely, a few who had not previously attended a vaccination consultation thought that this would be the only thing discussed.

“*I went in with the expectation that because my appointment was for the vaccination, that was possibly all we were going to be able to talk about.*”

[Owner 2]

Experienced owners had learnt that the vaccination consultation would focus on a general health check, but still identified their pet receiving the vaccine as the main reason for attending the appointment. Very rarely, owners mentioned the health check in relation to the value for money of their consultation; the majority discussed finance only relative to the cost of the preventative medicine products.

“*I do like to have that check. I think it’s good that they ask questions ‘cos it might be something that you haven’t thought to ask or if they just go through the basics. I think, it generates a conversation, I suppose…. and you do pay a bit of money for it as well to be quite honest. You do pay for your consultation and the vaccination so I think it’s worth getting their money’s worth as well, I think.*”

[Owner 9]

Despite not seeing it as the main reason for attending a vaccination consultation, almost all owners anticipated and expected that their pet would receive a full clinical examination or health check. This was thought to be an opportunity for the veterinary surgeon to identify any new health problems and to check any existing problems. However, owners’ expectations of what the examination would reveal differed widely. Broadly, owners of new pets and those with little pet owning experience described the examination as an important opportunity to find out if their pet was healthy, as they did not have the knowledge to identify health problems themselves. Several of these owners described these examinations as “reassuring” and had a high level of trust that the veterinary surgeon would find any problems that existed.

“*It gave us a starting point to know what his health was like, whether he needed to put on weight, whether he was like… if he had any problems that we couldn’t see. I mean he might have a skin problem that we can’t see because of his fur. And also because this is our very first pet, the vet could give us advice.*”

[Owner 2]

In contrast, several experienced owners with a high level of confidence in their own knowledge of pet health thought that they would have recognised any problems in their pet and would book a specific appointment with a veterinary surgeon if one was needed. These owners did not appear to value the clinical examination in the same way, as they anticipated that the veterinary surgeon would not find any problems of which they were unaware.

“*I think I’d… I mean I’m glad that they check them over, but I think I would trust my instincts to know if my pet… you know, because I’m quite fastidious if something’s not right with them, I would take them straight in. So I’d rather… I tend to think I would know if something is up with my pet.*”

[Owner 4]

However, all owners expected that they would be told about every problem identified during a clinical examination. None identified any reason why a veterinary surgeon might not mention something to them during a consultation. As a result, several assumed that if a veterinary surgeon did not discuss something, it was not abnormal or concerning.

“*I haven’t [discussed diet] with our [vet] and to be honest, they haven’t sort of said, oh, they’re overweight or they’re underweight or anything like that …. I think they have done with my sister’s cats when, erm, they were slightly overweight so I think they’d have mentioned it.*”

[Owner 9]

A few less experienced owners thought that the veterinary surgeon would be the best source of advice about how best to look after their pet, including what to feed them and how to prevent any illness, and had anticipated that this would also be discussed during the vaccination consultation. Other owners, typically those with older animals and more experience, felt it was their responsibility to ask the veterinary surgeon if they had specific questions but used a range of other information sources such as the internet for pet health advice. Several expressed uncertainty as to whether specific topics were relevant for discussion at a vaccination consultation.

“*I’ll be honest—I’ve had a look [online] but at the end of the day [my dog]’s too important to me to trust what I read on the internet. I’d rather hear it from a vet.*”

[Owner 11]

### 3.2. Veterinary Surgeons

Veterinary surgeons’ perceptions of their influence on the content of a vaccination consultation depended on the age of the animal involved. Puppy and kitten (primary) vaccination consultations were typically allocated longer periods of time than adult pet vaccination consultations and almost all described a practice protocol for what should be covered during these primary vaccination consultations. Few appeared to anticipate finding significant health problems on clinical examination in young animals, allowing them to follow the protocol fairly closely during the consultation. Many perceived this to be very useful, though some thought it difficult to cover in the time available.

“*We have our Pup Start pack so if it’s a puppy they kind of have a plan basically in place which is open to individual interpretation but there is a kind of set of reminder things you are supposed to cover… It is nice to have a structure there in place and there’s a lot to check to make sure you’ve gone through everything. Whilst it is nice to have that, it is difficult to cover it in 15 minutes*”.

[Veterinary surgeon 13]

The described structure and content of these primary vaccination consultations was remarkably consistent between veterinary surgeons. Typically, they were described to include a strong emphasis on establishing owners’ prior knowledge and educating them on diet, socialising, grooming and some aspects of traditional preventative healthcare such as the importance of worming and vaccinating. Several identified clear differences between the content of this consultation and vaccination consultations for older pets.

“*Yes, like, the puppy consult is different, we talk about socialisation, maybe neutering this time and microchip obviously, insurance so yes the puppy and kitten first vaccines are very different…*”

[Veterinary surgeon 11]

In contrast, all veterinary surgeons described the main focus of adult pet vaccination consultations to be soliciting owners’ concerns about their pet’s physical health and identifying additional health problems on clinical examination. This often appeared to result in a bespoke consultation that needed to be structured around each individual pet-owner combination.

“*Generally the first thing I ask is if they’ve got any concerns and if they haven’t got any concerns, then I’ll tend to ask the same set of questions. Is there any sickness and diarrhoea? Are they eating and drinking okay? Any coughing or sneezing? Any fits or faints or collapses or any lumps and bumps? Any lameness or stiffness, especially if they are older animals…*”

[Veterinary surgeon 1]

Only one veterinary surgeon described a practice-level checklist on which to structure these consultations; others thought such a structure was unnecessary or un-workable due to the variability in topics discussed. Several reported that during their clinical examination, they payed particular attention to specific body systems in which they had a personal interest.

“*I mean I talk about obesity all the time. That is a big one, I have thought about that, yes it is a big bug bear of mine so I really do go on about that…*”

[Veterinary surgeon 11]

In younger to middle-aged animals where health problems were rarely identified, vaccination consultations were described as quick and fairly routine. However, contrary to many owners, veterinary surgeons expected their clinical examination to identify new health problems in the majority of older pets, irrespective of their owners’ experience level. Cutaneous masses, obesity, dental disease and osteoarthritis were listed in this context in relation to older dogs, whilst weight loss, dental disease and excessive thirst were commonly mentioned in relation to older cats.

“*More than half the time, in fact over half the time, I’ll be discussing dentition and dental disease. Generally in cats I’ll be discussing obesity or weight loss, it’s usually one or the other. There are certain things that you have almost a prepared a speech on…*”

[Veterinary surgeon 2]

Many veterinary surgeons commented that owners would not look in their pets’ mouths, would allow their pets to gain too much weight or would not recognise signs of osteoarthritis. Often the veterinary surgeons suggested these problems presented a more serious welfare concern to the pet than any problem identified by their owner.

“*Repeatedly it will be teeth. Simply because it’s something that owners don’t see and that’s similar across the board of cats and dogs and the predominantly older group its lumps and bumps, but again, owners maybe haven’t felt because it’s been under the dog’s coat.*”

[Veterinary surgeon 10]

Very few discussed any potential educational role the veterinary practice could, or should, play in resolving this. For example, veterinary surgeons seldom described instigating discussions about how to recognise or prevent conditions described as common in older pets during adult pet vaccination consultations before these conditions arose. However, a few individuals felt this was extremely important. Those veterinary surgeons described using leaflets, follow-up telephone calls and repeat examinations to ensure that owners had understood the key messages. All described time pressures associated with this increased effort, and several described sacrificing their own break times to extend consultations for this reason.

“*So it is, you know I am pushed for time but I think it is important, I want to do the best for all the animals and all the clients. So I just try to do things as quickly as possible or even I have a little notebook where I think right okay if I haven’t got time to print things out now then I’ll do it later…*”

[Veterinary surgeon 14]

In contrast, other veterinary surgeons did not seem to think education to be such an important part of the vaccination consultation. Some alluded to a belief that educating owners was not a good use of the short consulting time available, often recalling experiences with owners who they felt were disinterested in being given advice. This latter group of veterinary surgeons also articulated a belief that experienced owners would know how to look after their pets so would not need advice.

“*I usually find out whether the person has had a pet before and tailor it to how much information they need basically. If they need advice on diet, need advice on worming, need advice on washing pets, yes it is depending on whether or not the person has had a pet before.*”

[Veterinary surgeon 13]

Interestingly, behavioural problems and diets were consistently mentioned as health topics that some veterinary surgeons would prefer not to discuss in adult pets, despite seemingly being comfortable to discuss these topics during primary vaccination consultations. Lack of time, poor confidence in their own knowledge and an expectation that owners would ask if they had a problem were reasons provided for avoiding these topics.

“*I think I am very wary of offering too much advice, especially if the pet is aggressive. I think I would be careful about what I advised in case it is the only advice they seek. Certainly if people were having problems I’d certainly point them in the direction of animal behaviourists who have day-to-day experience of dealing with it…*”

[Veterinary surgeon 12]

Perhaps due to the focus on health problems, time discussing preventative medicine products and protocols with owners during adult pet vaccination consultations appeared often to be limited to asking owners whether they were up-to-date with flea and worm treatments. Pet healthcare plans were mentioned by several as a huge benefit in relation to preventative medicine products. Advantages were framed both in terms of their own time management and owner compliance with preventative medicine administration.

“*I think again the pet health club service that we have is good because I know they’re already on Advocate so I can say do you need anything more today or reception will have already asked ‘Do you need anything more?’ so that’s covered. The pet health club does take a lot away actually and it does make things more efficient I think because they are already on it.*”

[Veterinary surgeon 3]

When asked, veterinary surgeons were unsure what owners expected from vaccination consultations but thought they should be expecting the focus to be on the health check. However, none of the veterinary surgeons described their practice providing any information for owners about what to expect beyond the description of the consultation as a “booster and health check”.

“*I use [the adult pet vaccination consultation] as a recheck as well if you’ve got anything that you’re concerned about or have been concerned about, but largely the vaccination in the vaccination appointment isn’t too important to me at all. It’s just something I’ll do on the way out.*”

[Veterinary surgeon 2]

Owners were thought to have differing levels of knowledge about pet health, and engagement in the consultation. Several veterinary surgeons identified that for this reason, it was much easier to vaccinate the animal of an owner that they knew, but few described this as routine. The challenges associated with modifying their consultation content and style to each individual owner-pet partnership were evident.

“*Each animal is different, do you know what I mean? And also the relationship you have with the clients are different. Some of them are very much, you know, I want to be in and out quickly and some of them will do the, ‘Oh while I am here can I just check this, that and the other’. You know, so it all very much depends with both of them…*”

[Veterinary surgeon 14]

## 4. Discussion

This is the first study to look at whether the content of vaccination consultations matches the expectations of owners and veterinary surgeons in the United Kingdom. These interviews identify that the preferred and anticipated balance of preventative healthcare, health assessment and education within a vaccination consultation differed within and between groups. Whilst primary vaccination consultations appear to have a clear and fairly consistent identity with a focus on preventative healthcare, the content of adult pet vaccination consultations appears much more diverse with variability in content driven by pet, owner and veterinary surgeon factors. These data suggest this lack of clear focus for the adult pet vaccination consultation may have negative consequences for client and veterinary surgeon satisfaction, and perhaps for animal welfare. This study will be of value to veterinary surgeons, practice managers and pharmaceutical companies who all have a role in shaping the pet vaccination consultation. 

Interventions to improve the vaccination consultation experience have been discussed for at least 20 years, yet little apparent progress has been made. In 1998, Coutts [7] suggested clients should be informed about what was likely to be discussed during vaccination consultations for pets of different ages, and the use of checklists has been proposed as an aid to standardising their content [26,27]. However, the complexity of factors that contribute to diversity identified in the current study suggest that any single intervention is unlikely be adequate. First, consensus must first be reached on the remit of the adult pet vaccination consultation. The divergence in opinion between owners and veterinary surgeons about what should be included suggests that both groups should be involved in reaching a consensus. We propose a re-definition of preventative healthcare to include all preventable conditions, such as obesity, behaviour and dental disease. This would better reflect both the current content of these consultations, and ensure that both prevention and management of these conditions receive adequate attention. 

The perception that owner education about health, disease, behaviour and welfare are not being prioritised by small animal veterinary surgeons has led to criticism of the profession [28,29]. This study suggests that advice for educational components to be included all adult pet vaccination consultations [8,9,30,31] has not been widely adopted in the UK. Theoretically, owner educational interventions should improve the welfare of both owners and their pets by enabling pet owners to recognise conditions at an early stage. This should then reduce the number of conditions identified for the first time during vaccination consultations. Prevalence data about common conditions seen in dogs and cats [32,33,34] could be used to identify the key conditions for which educational strategies should be developed. However, we have identified significant barriers to introducing this content into preventative healthcare consultations. Time pressure in these consultations are a major challenge; its implications and potential solutions are discussed elsewhere [19], as are the current and potential roles of veterinary nursing and reception staff in owner education [35]. Gaps in veterinary surgeons’ own knowledge were evident, particularly in relation to behaviour. This supports the findings of Roshier and McBride [36,37] that veterinary surgeons may feel insufficiently skilled to deal with common behavioural problems. Owners may therefore need to look elsewhere for advice, with the internet being the logical next step. Veterinary surgeons have previously expressed concern about the quality of online pet health information [38], and similar concerns were voiced by both veterinary surgeon and owner interviewees. Investment in reliable, centralized, online veterinary information for owners is long overdue. Its provision would provide an invaluable adjunctive resource to supplement practice-based education. 

Both veterinary surgeons and owners described a range of attitudes in their own responsibility towards animal welfare. Similar findings have been identified in studies examining horse owner attitudes to colic [39], owner attitudes to making decisions about chronically ill dogs [22,40] and farmers’ attitudes to lameness in cattle [41]. The diversity of veterinary surgeons’ personal professional identities [42] and limited moral reasoning ability [43] may be additional important factors in this. There is a real risk to animal welfare if a veterinary surgeon and an owner each abdicate primary responsibility to the other without adequately communicating this. Conversations to ascertain prior knowledge and to establish a trusted partnership of care are likely to be important [37,44,45,46]. Veterinary surgeons and owners identified benefits of achieving continuity of care across vaccination consultations, yet both groups suggested this was difficult to achieve. Prioritising continuity of care is likely to be an important component in strengthening relationships between veterinary surgeon and owner which are likely to benefit animal welfare.

These interviews provide an important first step in exploring attitudes to this common consultation. As with any qualitative research, direct extrapolation of these findings beyond this dataset should be performed with caution. Male pet owners and cat owners were particularly challenging to recruit, but all other aspects of both sampling frames were covered. Variability in owner attitudes towards veterinary practices by region of the UK is not reported, so the implication of involving owners predominantly from northern England in this research is unknown. Identifying the point at which data saturation has been reached is challenging [25]. Additional interviewees may have identified further important perspectives but we found respondents were fairly consistent in their expectations so it unlikely that new themes would have been identified. As all those involved in the collection and analysis of data were veterinary surgeons, there is the possibility that this may have biased the data collected and the coding of the data to develop themes. Analysis by researchers from other backgrounds may have presented these interviews using different themes but since this research was conducted with a veterinary audience in mind we do not consider this to be a significant limitation. 

Enrollment in the study required a degree of motivation by owners and veterinary surgeons. This may have naturally selected those participants with more polarised views. Our inclusion criteria selected for owners who had recently attended a practice for a preventative healthcare consultation but it is impossible to know how well participants were able to recall recent events or how accurately events were described. Furthermore, owners discussed a single recent vaccination consultation, veterinary surgeons discussed consultations in general, and this sometimes made drawing parallel conclusions from both interviews sets challenging. To overcome these challenges in future work, both veterinary surgeons and owners could be asked about the same consultation experience as previously done by Roshier and McBride [36,37], or consultations could be video-recorded [10]. However, these methods would be likely to logistically limit data collected to a smaller number of clinics, reducing the diversity in described experiences. 

## 5. Conclusions

These data form the first qualitative description of canine and feline preventative healthcare consultations. This research suggests that owners and veterinary surgeons may not share expectations about what will happen in a dog or cat vaccination consultation. We provide evidence for the potential value of providing information for owners about the likely content of their forthcoming vaccination consultation, encouraging continuity of care, and improving owner education about common preventable conditions. Redefining preventative healthcare to include all preventable conditions could have a positive impact on the welfare of owners and their pets, and on relationships between owners and veterinary surgeons. The role of the vaccination consultation in a wider animal welfare agenda has been further defined by this research and challenges associated with the current structure of adult pet vaccination consultations merit further attention. 

## Supplementary Materials

The following are available online at www.mdpi.com/2306-7381/5/1/7/s1, Table S1: Owner interview guide, Table S2: Veterinary surgeon interview guide, Table S3: Demographic data of owners interviewed, and their pets, Table S4: Demographic data of veterinary surgeons interviewed.
