# Supplementary material for: Owners and Veterinary Surgeons in the United Kingdom Disagree about What Should Happen during a Small Animal Vaccination Consultation
Source: Vet Sci. 2018 Jan 18;5(1):7. doi: 10.3390/vetsci5010007 (PMC5876577; doi:10.3390/vetsci5010007)
Supplement: Supplementary file 1 [file vetsci-05-00007-s001.pdf]

veterinary sciences

MDPI

Article

Owners and Veterinary Surgeons in the United Kingdom Disagree about what should Happen during a Small Animal Vaccination Consultation

Supplementary Materials:

Table S1. Owner interview guide

Each owner was asked the same broad questions; prompts were used at the discretion of the interviewer.

Question	Prompts
Can you tell me a little bit about the last time you took *PETS NAME* to the vets [for a preventative healthcare consultation]?	a. Why did you decide to make the appointment?<br>b. Why did you take *PETS NAME* in to see the vet?<br>c. Can you describe to me what happened during your visit to the practice?<br>d. How does that compare with what you were expecting before you went for the consultation?<br>e. So *PETS NAME* had a vaccination during the consultation (if applicable). What do you know about vaccination? What are your opinions about vaccination in general?<br>f. Was the vaccination your main reason for making the appointment?<br>g. Were there any other reasons why you decided to make an appointment?
Did you talk to the vet about anything else, other than the vaccination, during the consultation?	a. What sorts of things did you talk about?<br>b. What prompted these discussions? Did you bring these things up or did the vet?<br>c. What about other types of preventive care such as worm and flea prevention, did you discuss these with your vet? Why did you/did you not decide to discuss these with the vet? Have you discussed these previously with your vet? If so when?<br>d. Do you think these other types of preventive care are important?<br>e. Do you get information on preventive care from anywhere else? Why do you decide to get information/flea and worm treatments from these places?<br>f. Did you talk to the vet about any other aspects of *PETS NAME* health? Can you tell me a bit about those discussions?<br>g. Did you talk to the vet about anything related to your *PETS NAME* everyday care, for example, diet and exercise? Can you tell me a bit about those discussions?<br>h. Did you talk to the vet about anything related to *PETS NAME* behaviour during your visit to the practice? Can you tell me a bit about those discussions?<br>i. Did you find it useful to talk to your vet about these other things?

	j. Some previous research has suggested that vets and owners frequently talk about lots of different areas of pet health during vaccination consultations. How does this fit with your experience?<br>k. Did you talk to any other staff at the practice about *PETS NAME* for example vet nurses or receptionists? What sorts of things did you discuss? Who prompted the discussion? How did the discussions with the nurse/receptionist compare to the discussion you had with the vet?
Is there anything you can think of that would have made your visit to the practice more beneficial to you and *PETS NAME*? Why do you think this would have been beneficial?	a. Overall do you think your visit to the practice was beneficial? Did you get everything out of it that you were expecting to?<br>b. Was there anything about your visit to the practice that would make you reluctant to bring *PETS NAME* in for an appointment next time? Why would this make you reluctant?<br>c. Was there anything else you would have liked to talk to the vet about?<br>d. How about the time available to the consultation? Do you think the right amount of time was available?<br>e. Some people use a 'checklist' or questionnaire for owners to complete before the consultation. How do you feel about this idea? Do you think this would be practical? Do you think this would be useful?<br>f. Some practices involve nurses in the consultation with the nurse often starting the consultation then the vet coming in part way through? Have you any experience of this? How would you feel about this?<br>g. Have you ever taken *PETS NAME* to the practice for a general check over, when they were otherwise healthy and not due their vaccinations? (if yes) Did you find this useful? Why/why not? (If no) How would you feel about doing this? Why?
Is there anything else that we haven't discussed on the subject of preventive care that you would like to add?	

Table S2. Veterinary surgeon interview guide

All veterinary surgeons were asked the same questions; prompts were used with the discretion of the interviewer.

Question	Prompts
Could you tell me a little bit about how your 'typical' vaccination consultation would proceed? Could you tell me a bit about why you tend to conduct your consultation in this way?	a. How about history taking? What do you typically ask during the consultation?<br>b. How about the clinical examination? How do you conduct this? Why do you conduct it in that way?<br>c. How about for different types of patients – boosters vs primary course, cats vs dog – do you do things differently?<br>d. Do you see ever see patients presented for 'general health checks' i.e. healthy patients not due a vaccination? Do you feel these consultations are worthwhile? How do you think owners see these consultations?<br>e. Do you have any practice protocols or guidelines around preventive medicine? Could you tell me a bit about those? Do you find them useful?<br>f. Why do you think owners bring their pets in for a preventive health consultation?
Do you ever discuss any other aspects of health with pet owners during preventive medicine consultations? Could you tell me a little bit about your experience of these discussions?	a. What other aspects of health do you commonly discuss? Why do you think these aspects of health are discussed most commonly? What prompts these discussions? Do you bring them up or does the owner? Do you think there are benefits in discussing other aspects of health during preventive medicine consultations?<br>b. How do owners react to these discussions? Do you think they find them useful?<br>c. How about general everyday pet care such as diet and exercise. Do you ever discuss these things during the consultation?<br>d. Tell me a bit about routine parasite prevention. Is this something you discuss with clients during vaccination consultations? Can you tell me a little bit about these discussions? How do you think pet owners view these treatments?<br>e. How about pet behaviour. Is something you ever discuss? What types of things do you discuss and what prompts these discussions?<br>f. Do you think there are benefits in discussing other aspects of health during preventive medicine consultations?
If you had unlimited time and resources, is there anything you would do differently in these consultations?	a. Do you think these consultations are useful? Is there a way we can make things better for the pet owner and patient?<br>b. How about from your point of view. Is there anything that could be done to make the preventive medicine consultation a better experience for you as a vet, or to improve your job satisfaction?<br>c. Some practices use nurses during the vaccination consultation, for example the Safari system. Is this something you have any experience of? How would you feel about this? Would it be work in your practice?<br>d. Have you ever asked owner's to complete a questionnaire about their pet's health prior to their consultation? If so, how did this work? Is this something you think would be useful? What do you think owners would think about this?

	e. Have you ever used a formal 'checklist' to structure your consultations? How would you feel about using something like this?<br>f. How about the time allocated for the consultation? Do you feel as if you have long enough? If you had longer appointments, how would you use the extra time?
Is there anything else that we haven't discussed on the subject of preventive care that you would like to add?	

Table S3. Demographics of owner interviewees and of their pets discussed during the interviews.

Owner ID	Sex	Owner employment status and job type	Owner location	Owned pets previously?	Veterinary practice type attended	Demographics details of pets currently owned
1	Female	Working, healthcare	North England	No	Independent	4 year old male neutered Lhasa Apso dog
2	Female	Working, undisclosed non-science job	Scotland	No	Independent	2 year old female neutered Goldendoodle dog
3	Female	Working, animal care	North England	Yes	Independent	3 year old female neutered Dachshund dog<br>3 year old female neutered Poodle cross dog
4	Female	Working, undisclosed non-science based job	North England	Yes	Independent	3 year old female neutered domestic shorthaired rescue cat
5	Female	Working, scientist	Midlands	Yes	Independent	11 year old male entire working Cocker Spaniel gundog<br>6 year old female entire working Cocker Spaniel gundog<br>2 year old female entire working Cocker Spaniel gundog
6	Female	Working, undisclosed non-science job	North England	Yes	Independent	8 year old female neutered Bedlington terrier rescue dog
7	Female	Working, customer service	North England	Yes	Independent	8 year old female neutered Goldendoodle dog<br>3 year old female neutered Goldendoodle dog<br>9 year old female entire toy Poodle dog
8	Female	Working, undisclosed non-science job	Midlands	Yes	Independent	8 year old male neutered Cockapoo agility dog<br>7 year old female neutered Cockapoo agility dog
9	Female	Working, undisclosed non-science job	North England	No	Independent	6 year old female neutered domestic shorthaired cat<br>5 year old male neutered domestic longhaired cat<br>3 year old male neutered Chihuahua dog
10	Female	Retired	North England	Yes	Independent	4 month old male entire Cockapoo dog
11	Female	Working, undisclosed non-science job	North England	Yes	Independent	7 year old male neutered Cavalier King Charles Spaniel dog
12	Female	Working, child care	North England	No	Corporate	6 month old female entire working Cocker Spaniel dog
13	Female	Retired	North England	Yes	Independent	2 year old male neutered Lurcher dog
14	Female	Working, animal care	North England	No	Corporate	5 year old male neutered Labradoodle dog<br>4 year old male neutered Labradoodle dog
15	Female	Retired	North England	Yes	Corporate	2 year old female neutered crossbreed dog<br>10 year old male neutered West Highland White tTrrier dog

Table S4. Demographic details of veterinary surgeons interviewed.

Veterinary surgeon ID	Sex	Year of graduation	Veterinary education undertaken in the UK?	Position within practice	Practice location	Practice description
1	Female	2004	Yes	Senior vet	North England	Small animal, independent, multi-branch
2	Male	2015	Yes	Assistant	North England	Small animal, independent, multi-branch
3	Female	2008	Yes	Assistant	Wales	Small animal, independent, multi-branch
4	Female	2003	Yes	Assistant	South East England	Mixed, independent, multi-branch
5	Female	2000	Yes	Clinical director	South England	Small animal, corporate, multi-branch
6	Female	2001	Yes	Assistant	South East England	Small animal, corporate, multi-branch
7	Male	2006	No	Clinical director	South East England	Small animal, corporate, single branch
8	Male	1988	Yes	Partner	Midlands	Small animal, independent, single branch
9	Female	2003	No	Clinical Director	Midlands	Small animal, corporate, single branch
10	Female	2004	Yes	Assistant	Scotland	Small animal, independent, multi-branch
11	Female	2005	Yes	Assistant	North England	Small animal, independent, multi-branch
12	Male	2008	Yes	Assistant	North England	Mixed, independent, single branch
13	Female	2008	Ireland	Assistant	Scotland	Small animal, independent, multi-branch
14	Female	1993	No	Assistant	South West England	Small animal, independent, multi-branch

© 2018 by the authors. Submitted for possible open access publication under the terms and conditions of the Creative Commons Attribution (CC BY) license (http://creativecommons.org/licenses/by/4.0/).
